# Regional Influences on Chinese Medicine Education: Comparing Australia and Hong Kong

**DOI:** 10.1155/2016/6960207

**Published:** 2016-06-09

**Authors:** Caragh Brosnan, Vincent C. H. Chung, Anthony L. Zhang, Jon Adams

**Affiliations:** ^1^School of Humanities and Social Science, Faculty of Education and Arts, University of Newcastle, University Drive, Callaghan, NSW 2308, Australia; ^2^Australian Research Centre in Complementary and Integrative Medicine, Faculty of Health, University of Technology Sydney, 15 Broadway, Ultimo, NSW 2007, Australia; ^3^The Jockey Club School of Public Health and Primary Care, The Chinese University of Hong Kong, Prince of Wales Hospital, Shatin, New Territories, Hong Kong; ^4^Health Sciences, RMIT University, P.O. Box 71, Bundoora, VIC 3083, Australia

## Abstract

High quality education programs are essential for preparing the next generation of Chinese medicine (CM) practitioners. Currently, training in CM occurs within differing health and education policy contexts. There has been little analysis of the factors influencing the form and status of CM education in different regions. Such a task is important for understanding how CM is evolving internationally and predicting future workforce characteristics. This paper compares the status of CM education in Australia and Hong Kong across a range of dimensions: historical and current positions in the national higher education system, regulatory context and relationship to the health system, and public and professional legitimacy. The analysis highlights the different ways in which CM education is developing in these settings, with Hong Kong providing somewhat greater access to clinical training opportunities for CM students. However, common trends and challenges shape CM education in both regions, including marginalisation from mainstream health professions, a small but established presence in universities, and an emphasis on biomedical research. Three factors stand out as significant for the evolution of CM education in Australia and Hong Kong and may have international implications: continuing biomedical dominance, increased competition between universities, and strengthened links with mainland China.

## 1. Introduction

As Chinese medicine (CM) has spread throughout the world, it has been absorbed, interpreted, and transformed within different national contexts [1–4]. A key channel through which such processes occur is in the training and education of CM practitioners and the intersection of CM training with broader national education systems. It is often via education that particular philosophies and practices come to predominate and are adopted by the next generation of practitioners. Critically analysing CM education in different national settings can therefore provide useful insights into how and why CM is evolving internationally. This discussion paper compares the status of CM education in Australia and Hong Kong in order to understand how these two contrasting national settings shape CM education and, in turn, the future CM workforce in each region.

CM practice in both Australia and Hong Kong is statutorily regulated. The Chinese Medicine Board of Australia (CMBA) and the Chinese Medicine Council of Hong Kong (CMCHK) are responsible for implementing regulations on CM practice, respectively, for the two regions. Obtaining licensure for practice from either regulatory body requires professional education in accredited programs. Unlike many other English-speaking jurisdictions, such programs are offered in public universities in both regions. The design of these 4–6-year programs invariably illustrates reference to the CM curriculum in mainland China, of which 60–70% of the content is focused on CM, and the remaining curriculum is focused upon biomedicine. In Hong Kong, the CM component is taught in Chinese, while the biomedicine component is often taught in English. In both Hong Kong and Australia, mandatory continuing medical education/continuing professional development (CME/CPD) for registered CM practitioners is in place and fulfilment of relevant requirements is necessary for revalidation. The majority of graduates from CM programs in both regions will practice in the private sector, providing outpatient services in either solo or group practice.

Despite these similarities, these programs operate in different cultural and health system contexts. In Hong Kong, the development of CM services has become a constitutional mandate after reunification of China. Limited outpatient CM services are provided in the tax-funded healthcare system and pilot programs on CM inpatient services within public hospital are in progress. While CM education has gained top-down legitimacy from the government, acceptance of CM graduates by conventional medical practitioners remains limited and there has been little interprofessional collaboration. In Australia, the only form of CM available through the public health system is acupuncture, where it is carried out by a medical doctor. Access to CM in Australia therefore involves out-of-pocket expenses, although costs may be subsidised by some private healthcare funds, with nearly 60% of the adult population having private health cover in Australia [5].

Given these similarities and differences, Australia and Hong Kong constitute excellent case studies for examining how wider historical and policy contexts may shape discussion and subsequent development of CM education in health systems where biomedicine dominates.

## 2. Materials and Methods

A small number of prior studies have examined the influence of culture and social structures on CM education in the United States [1, 3], United Kingdom [6], and Australia [7, 8]. However, there is a distinct lack of cross-national comparative research in this area, especially when considering the broader context of education in each country beyond specific degree programs. Here, we aim to broaden the analytic frame by taking stock of different influences on the form that CM education takes in two regions with distinct but overlapping regulatory contexts, organisational structures, and health system features.

Drawing on available literature, the paper first provides a brief overview of the historical position of CM in the Australian and Hong Kong education systems, before going on to examine CM's current position and the shifts taking place in higher education, the regulatory context and place of CM in the healthcare system, and the cultural legitimacy of CM in each region. Consideration is also given to the reasons for the similarities and differences in the status of CM education between Australia and Hong Kong. The paper then draws the key issues together to provide an overall assessment of the constraints and opportunities for developing CM education in the future in Australia and Hong Kong and considers the implications for other similar regions where CM is taught. The conclusion highlights the importance and relevance of this research, especially in terms of future investigations into CM education worldwide.

## 3. Results

### 3.1. Historical Position of CM in the National Higher Education System

While the history of CM in Australia is vastly different to that of Hong Kong, historical events have nevertheless impacted CM's current position within the higher education systems of both countries.

#### 3.1.1. Australia

Though still considered complementary or alternative to mainstream biomedicine, CM, including acupuncture and herbal medicine, has been well established in Australia since the gold rush era of the 1850s [2, 9]. Increased Asian migration to Australia in the late twentieth century was accompanied by a significant increase in CM use [9]. CM formal education has historically been skewed towards acupuncture, with the first (private) acupuncture colleges opening in the 1970s [10] (offering diplomas), prior to which some acupuncture training had been offered in chiropractic or naturopathic colleges [11]. Bachelor degree programs were established or transferred from colleges into four publicly funded universities from the early 1990s, with the first degree program covering both acupuncture and herbal medicine opening at the Royal Melbourne Institute of Technology (RMIT) in 1996 [10]. This move into the university system occurred well before the statutory regulation of CM practice in Australia and was seen as offering opportunities for greater access to research and teaching resources [7].

#### 3.1.2. Hong Kong

As a tradition of China, CM has been used for centuries and it has been officially included in the healthcare system in mainland China since the 1950s [12]. With the government's consistent support, CM remains a key part of China's health service today [13]. Nevertheless, CM in Hong Kong has followed a very different path, due to Hong Kong's status as a British colony from 1841 to 1997. In the early colonial days, the local Chinese community considered CM as their main form of healthcare. Tung Wah Hospital was the first CM Hospital in Hong Kong, opening in the late nineteenth century. It made a significant contribution to the provision of basic healthcare in the Chinese population during that period [14]. However, following World War II, a tax-funded healthcare system was established with biomedicine being the exclusive type of healthcare and the role of CM largely sidelined to the private sector, often with CM practitioners working in solo practice. The colonial government regarded CM as part of the “Chinese cultural custom” instead of a formal healthcare modality [15]. Instead of the Secretariat for Health, CM came under the administrative purview of the Secretariat for Home Affairs [16].

The marginal status of CM was also reflected in legislation relating to healthcare. The colonial Medical Registration Ordinance specified that only biomedical clinicians were subject to regulation, and the practice of CM was considered to be out of scope [17]. Due to lack of regulation, tertiary education was not a prerequisite for practice in CM. Apprenticeships with family members or “masters” were the usual pathway to a CM career, often supplemented with lecture-based training provided by CM associations with mixed quality [18]. In the early 1990s, CM education first appeared in the School of Professional and Continuing Education, University of Hong Kong. However, despite its appearance in the tertiary education sector, CM remained marginalised. Only conventional clinicians were allowed to use the title “doctor,” and this rule continues today even after regulation. Sharing clinics between biomedical and CM clinicians was prohibited and the latter had no rights in issuing death, sick leave, or health status assessment certificates and were forbidden to use any “biomedical” instruments like syringes and stethoscopes [19]. These regulations led to the creation of a formal medical system based only on biomedicine.

This situation changed with the reunification of Hong Kong and China on 1 July 1997, as the constitutional law of the then newly established Hong Kong Special Administrative Region (SAR) mandated the development of CM in the territory [20]. Under this policy initiative, Hong Kong Baptist University launched the full-time, five-year bachelor degree in CM in 1998: the first of its kind after reunification with China. Similar to Australia, the establishment of a School of Chinese Medicine within a public university was considered a milestone for research and learning in CM in postcolonial Hong Kong [21].

### 3.2. Current Higher Education Context and the Position of CM

Differences exist between the current positions of CM in Australian higher education in comparison with Hong Kong, yet there are also similarities in terms of research and funding.

#### 3.2.1. Australia

Despite having been represented within Australian universities for over two decades, Garvey [7] describes CM as just “one tiny fish in a very large tertiary education…pond” (p. 7). Indeed, only three of the 40 Australian universities currently teach CM (RMIT University, University of Technology Sydney, and University of Western Sydney). All now offer qualifications in both acupuncture and herbal medicine, and a range of four-/five-year bachelors and three-year (part-time) Masters programs are available. Entry requirements into these courses include an Australian Tertiary Admission Rank (ATAR) in the 70s–80s (out of 100), which is higher than the average ATAR of around 70 [22] and means CM university programs are more competitive to gain entrance to than nursing degrees, but less so than medicine. In addition to universities, 4-year CM bachelor degree programs are also offered at three private colleges (Endeavour College of Natural Health, Southern School of Natural Therapies, and Sydney Institute of Traditional Chinese Medicine).

National enrolment figures are not published regularly. A 2010 study reports 144 final year students across the (then) seven institutions [23] although national registration in 2012 may have seen these numbers expand. The profession itself is also relatively small, but growing, with just under 4500 practitioners registered in mid-2015 [24]. This compares to over 100,000 conventional medical doctors currently registered in Australia [25], representing a practitioner : population ratio of 1 : 232 for conventional medicine versus 1 : 5314 for CM.

As a minor player, CM is subject to shifts affecting the Australian higher education sector as a whole, including cuts in public funding and universities' increased reliance on student fees and external research grants. Currently under discussion in Australia are policy changes that would see universities permitted to charge uncapped fees for courses and increased public funding for private education providers. CM is unusual among complementary medicine and other disciplines in Australia in that CM degrees are already offered both by universities and by private colleges. The proposed deregulation of university funding in Australia would see increased competition for students from private colleges, which may be able to undercut universities' fees, potentially impacting CM's position in the university sector. The case of naturopathy is informative here: it gradually disappeared from Australian universities after new funding schemes for private education were introduced in 2006 [26].

At an international level, the opening of higher education markets and a new emphasis on competitive ranking systems have affected CM alongside all other university disciplines [27]. Recent global competition centres on research funding and outputs and within universities disciplines are increasingly evaluated against these metrics, with natural and medical science disciplines typically coming out on top [28]. In Australian universities, complementary medicine disciplines have struggled to keep pace with this research environment, although among them CM has had the greatest success, with a number of competitive public research grants awarded in CM in the past decade [29]. However, CM research is not necessarily recognised as such in the national research assessment exercise, the “Excellence in Research for Australia” (ERA). Within the ERA, there is a single category for “complementary and alternative medicine” as a research field, and many of the CM studies conducted are counted within the “clinical sciences” or “pharmacology and pharmaceutical sciences” categories, thereby masking the actual research strength of CM in Australia. This merging of CM with other disciplines may also reflect a trend towards the biomedicalisation of CM. Indeed, funding is typically awarded for research that fits within a biomedical paradigm, focussing on molecular biology or employing randomised-control trials [7]. Such funding success may represent a double-edged sword for CM, with some commentators raising concerns over the fate of CM's traditional concepts which are not easily included in such research frameworks [7].

Funds from the Chinese Government and Chinese pharmaceutical companies have also provided important resources for CM research in Australia, and many universities and private colleges are affiliated with Chinese institutions [30]. The relationship between research and education in CM within Australia is not straightforward however, and recent years have seen substantial research funding for complementary medicine directed towards universities or university centres that do not necessarily teach it, for example, the establishment of the Zhendong Australia-China Centre for Molecular Traditional Chinese Medicine, University of Adelaide, and the Australian Research Centre in Complementary and Integrative Medicine (ARCCIM), University of Technology Sydney. While some of these centres promote collaborations between research and clinical practice/practitioners, the potential increased privatisation of higher education in Australia may result in a deeper split between complementary medicine research and teaching.

#### 3.2.2. Hong Kong

In Hong Kong, it has been 17 years since the first batch of full-time students enrolled in an undergraduate CM program offered by a public university. While there are three Schools of Chinese Medicine in the territory (Chinese University of Hong Kong, Hong Kong Baptist University, and University of Hong Kong), the scale of CM undergraduate education has remained small, with total new enrolment of about 100 per year [31]. Entry requirements from high school are similar to those for nursing degrees and lower than those for medicine [32–34]. The curriculum at all institutions was designed according to the accreditation requirement from the CMCHK, covering Chinese herbal medicine, acupuncture, and bone setting [35]. The degree programs have now extended to 6 years. Despite such small intakes, the CM workforce is not small as many practitioners have been able to obtain registration via grandfathering processes. By 2015, there were 6,898 registered Chinese medicine practitioners on the CMCHK list [36]. This translates to a CM-practitioner-to-population ratio of 1 : 1053, significantly higher than in Australia. The conventional doctor : population ratio is 1 : 541 [37], but since CM only constitutes about 20% of all outpatient care provision in Hong Kong [38], there is a slight oversupply of CM practitioners.

This risk of oversupply is exaggerated by an increasing number of candidates sitting for the CMCHK licensing examinations. On top of local CM students, graduates from 31 recognized CM universities in mainland China are eligible to sit the examination and become registered CM practitioners in Hong Kong if they pass all requirements. Every year, more than one thousand Hong Kong high school graduates are admitted to mainland CM universities and the number is increasing. It is likely that they will return to Hong Kong and sit for the licensing examination [39]. Mainland CM universities are now in direct competition with the local CM programs for enrolment. The Hong Kong program is slightly disadvantaged as it is one year longer than the 5-year course provided across mainland China, and the fees are at least 4 times higher [40]. Another threat to the local CM programs is the relatively lower government funding as compared to other clinical subjects like conventional medicine and dentistry. Although all these programs are six years in length, public funding for CM is two times less, causing staff shortages in the local schools of CM [31].

With regard to research, schools of CM in Hong Kong face similar challenges to their Australian counterparts in maintaining competitiveness. A dedicated CM theme was established under the Hong Kong Health and Medical Research Fund in 2002, encouraging health services research and clinical trials on CM. However, with a cap of HKD$ 1 million (AUD$ 185,000) per project, only trials of modest size can be performed [41]. Another difficulty is that a very stringent requirement is set by the Department of Health on the use of Chinese herbal medicine in clinical trial settings. At the time of writing, there is only one Chinese herbal product approved for human trial, despite the fact that such herbs are widely used in the community already. Despite the government's attempt to develop Chinese medicine in an “evidence based” approach, the largest share of research funding is often granted to laboratory based research that does not inform clinical practice directly. Such funding is often channelled to departments that have no involvement in CM teaching. While the three Schools of Chinese Medicine were performing satisfactorily in the last research assessment exercise, it is uncertain how this may impact educational outcomes, as pedagogical research is minimal in all three schools. Policy directions on CM research and teaching in Hong Kong appear to be developed in an uncoordinated fashion and are not entirely concordant with the government's initiative in building an evidence base for CM practice [42].

### 3.3. Regulatory Context and Place in the Healthcare System

Similarities in the path towards statutory regulation of CM in Australia and Hong Kong reflect their British colonial histories, while CM's current regulatory status is indicative of government policies regarding both CM and biomedicine.

#### 3.3.1. Australia

In 2000, the State of Victoria, Australia, became the first western state in the world to establish statutory regulation of CM [10], which included the introduction of minimum education standards for the first time. This was followed in 2012 by the inclusion of CM in Australia's National Registration and Accreditation Scheme, where it joined 13 other health professions. This process led to the application of national education and competency standards to CM practitioners, including the requirement of a recognised degree qualification in CM to be able to register and practice. The degree programs themselves must be accredited by the CMBA in order for their graduates to be eligible for registration and each CM degree program is currently undergoing or has recently undergone this new accreditation process for the first time, placing a significant administrative and financial burden on CM education providers. Accreditation standards have been developed and tailored specifically for CM degree programs and include detailed requirements relating to the theory and practice of acupuncture and herbal medicine, basic understanding of Chinese language, and mastery of the Pin Yin system, as well as basic scientific competencies and more generic health professional learning outcomes relating to ethical conduct, communication, risk management, and so on [43].

Despite having joined the list of registered professions, CM practitioners remain largely excluded from publicly funded healthcare and hospitals in Australia and most CM practitioners operate as private businesspeople in the community. Rather than being integrated into the health system, CM students receive most of their clinical training in a single university- or college-based clinic, limiting their exposure to both clinical populations and presentations. Although it is common for Australian-based CM students to also complete a placement in China, the therapies, conditions, and clinical settings predominant in the Chinese health system do not necessarily apply to practice in Australia. Unlike many other health professions (e.g., medicine, pharmacy, and psychology), there are currently no formal supervisory or training pathways for CM graduates in Australia, aside from standard CPD requirements.

Interestingly, the Australian Government has recently signalled its support of CM through the signing of a letter of understanding with the Chinese Government in June 2015 (in conjunction with a new free trade agreement) agreeing to promote cooperation between the two countries around CM research and the recognition of qualifications [44] and the sustained Australian Government policy of increasing Australia's links to Asia may reap benefits for CM education.

#### 3.3.2. Hong Kong

Though now reunified with China, Hong Kong's regulatory situation for CM is very similar to Australia and continues to reflect the region's British colonial heritage. Despite statutory regulation and CM program accreditation, CM practitioners remain a “parallel” profession to conventional medical doctors as well as other healthcare professionals. While all CM programs include biomedicine components, exposure to CM in medical, nursing, and allied health education remains very limited. Interprofessional learning is yet to be scaled up, although, at undergraduate level, Schools of Chinese Medicine are providing basic CM education to medical, nursing, and pharmacy students. At postgraduate level, local universities also collaborated with the Hospital Authority in organising a CM training course for practising healthcare professionals. Since CM is not provided in all publicly funded hospitals and clinics, teamwork across CM and conventional medicine is rare. Currently, there is no formal mechanism for facilitating interprofessional referral between CM and conventional medical clinicians, and no publicly funded hospitals currently accept referral from private CM practitioners. Given the limited interaction between CM and biomedical clinicians, the two professions are considered “parallel”: in the public sector, CM practitioners are not subordinate to conventional clinicians, as CM provision is often provided in stand-alone clinics with very limited interprofessional referral mechanisms; and, in the private sector, there is no mechanism for interprofessional teamwork and therefore subordination does not exist.

The government has provided partial subsidy (20%) to CM outpatient services, which are comanaged in a tripartite mode by the schools of CM, nongovernmental organizations, and the Hospital Authority (the public healthcare service provider) on a predominantly self-financed basis. The first tripartite CM clinic was established in 2003 and currently there are 18 in the territory [45]. Patients' out-of-pocket payments are the main source of funding although quotas of fee waivers are reserved for those with financial difficulties. These tripartite clinics must balance between maintaining financial sustainability and serving as clinical training sites for CM students. They are also the main employer for local CM graduates, providing a structured training program over a three-year contract. While the contribution of tripartite clinics to training junior CM practitioner should be recognized, training quality may be compromised due to financial pressure [46].

CM hospitals in mainland China are alternative sites for clinical training for Hong Kong CM students. There, CM students' final year internship often takes place in environments where both CM and biomedical treatments are prescribed by the same clinician. However, as with Australian-based CM students, knowledge and skills gained from an integrative inpatient environment in China are not directly applicable to Hong Kong CM students' future role as a primary care clinician providing CM-only treatments in Hong Kong [47].

In Hong Kong, CME is mandatory for CM practice license revalidation but it is often viewed negatively by local CM graduates and repetition of undergraduate content in CME is common as such content is often geared to less well trained practitioners who previously received grandfathering licenses [21].

### 3.4. Public and Professional Legitimacy

While there are differences in terms of public use and acceptance of CM in Australia and Hong Kong, issues regarding its legitimacy within public medicine and tertiary institutions exist in both countries and primarily stem from the relationship between CM and biomedicine.

#### 3.4.1. Australia

Accompanying the uneven government support of CM practice and education in Australia are varying levels of acceptance among the public and other health professions. Acupuncture and Chinese herbal medicine are relatively commonly used, with 9.2% and 7% (resp.) of national survey respondents reporting usage in the previous 12 months [48]. However, CM is argued to lack a strong presence in Australia [7] as well as an identifiable peak professional body [30]. In terms of CM education, Garvey [7] has suggested that because regulated and accredited CM training remains a relatively new concept in Australia, the discipline will continue to be treated with scepticism by proponents of biomedicine and will need to “prove” its legitimacy as a healthcare practice.

Indeed, the same year in which CM was included in the national registration system saw a significant backlash against the teaching of complementary medicine in Australian universities. This campaign was led by the Friends of Science in Medicine, a lobby group primarily composed of academic doctors and scientists, who argued through the news media that complementary medicine, including CM, was “pseudoscience” that should not be taught in publicly funded universities [49]. Representatives of universities teaching CM and other types of complementary medicine responded to the campaign predominantly by asserting that such degree programs are in fact based on bioscientific foundations [49]. What this (ongoing) debate suggests is that the legitimacy of CM within Australian universities does hinge on its integration of bioscientific approaches.

#### 3.4.2. Hong Kong

While current support for CM education and service within the public sector is limited, usage of CM among the Hong Kong population is high with more than 60% of the general public having ever consulted a CM practitioner [50]. One territory-wide survey suggested that the prevalence of consulting a CM practitioner in the past year is around 20%. Within this 20%, 17% sought care from both CM and biomedicine clinicians and 3% only consulted CM practitioners [38]. The current Chief Executive of Hong Kong has shown strong support for the further advancement of CM in an “evidence based” manner, and in 2013 a Chinese Medicine Development Committee was established with the Secretary for Food and Health as Chairman [51]. Following recommendations from the Chinese Medicine Development Committee, three main policy initiatives have been announced in 2015 [52]. The first will be the establishment of a testing centre for Chinese medicines directly managed by the Department of Health and with a goal of setting up reference standards on safety, quality, and testing methods of Chinese herbal medicines. This centre will provide upstream assurance on the safe use of herbs. The other two policies are more service oriented. A site is reserved for the establishment of a new CM hospital in the territory, to provide inpatient care as well as teaching support. This is an entirely new initiative for the Hong Kong health system.

In order to explore feasible modes of operation, the third policy of piloting integrative Chinese biomedical clinical services in public hospitals was launched. Three pilot integrative care projects on cancer palliative care, low back pain, and stroke rehabilitation were launched and evaluation results will inform regulation and mode of operation of the future CM hospital. In these pilots, the CM treatments for all three conditions are mainly based on protocols that were designed by reviewing existing evidence and consensus between CM and biomedicine experts. Prescription flexibility of CM practitioners is limited and the essential feature of individualized treatment in CM is partly compromised. These three policy initiatives seem to suggest that the “biomedical standardization” of CM practice is key for acceptance in a healthcare environment dominated by conventional medicine. Unlike in Australia where CM's legitimacy is being directly challenged, in Hong Kong, the patterns tend to favour the assimilation of CM with a gatekeeping role for conventional medical clinicians and pharmacists. Recently, the Hong Kong Government has issued a call for Expressions of Interest from organizations that are keen to participate in the future operation of the Chinese medicine hospital [53]. These opinions may shape possible operational models and impact the interprofessional relationship between Chinese and conventional clinicians.

## 4. Discussion

The comparison of the factors impacting CM education in Hong Kong and Australia has revealed some striking similarities between the two regions, as well as important differences, highlighting the role of history, culture, and politics in the evolution of CM. CM was integrated into the formal tertiary education sector much earlier in Australia than in Hong Kong, first via private colleges and later also via universities. However, the reunification with China acted as a catalyst for the development of CM education in Hong Kong, and CM now has a comparatively larger presence in the university sector there, with three out of eight public universities teaching CM, compared to less than 10 per cent of universities in Australia. Postgraduate clinical training pathways are much better established in Hong Kong via the publicly funded CM clinics, while no such programs exist in Australia where CM remains truly excluded from the public healthcare system. However, rates of CM usage in Hong Kong are only two to three times higher than in Australia, while the practitioner : population ratio is five times higher, making oversupply of CM practitioners a greater problem in Hong Kong, where conventional medicine also predominates.

Beyond these differences, three key interrelated issues seem to stand out as being significant for the status of CM education in both Australia and Hong Kong. These key factors also potentially have ramifications for CM education in other regions outside mainland China. The first is the impact of ongoing biomedical dominance within healthcare systems. In both Hong Kong and Australia, this has limited the CM clinical training opportunities available at undergraduate and postgraduate levels and curbed the development of interprofessional education, now recognised as crucial in other health disciplines [54]. This situation is, in turn, likely to perpetuate CM's marginalisation, as other health practitioners' understanding of CM and ability to refer to CM practitioners will remain limited. Furthermore, the relatively low profile of CM in these regions, compared to conventional medicine, means that the scale of CM education has remained small, being represented in only 3 universities apiece in Australia and Hong Kong. As a university discipline, CM lacks the critical mass within these regions that is needed to develop a strong professional field, through holding local conferences, establishing cross-institutional collaborations, and so on.

This leads on to the second key issue we have identified, which is the impact that the global competition between universities (for students, status, and research funding) is having on CM education and may have in the future. University schools of CM in Hong Kong compete for students with the more affordable mainland universities, while Australian CM university departments compete with private colleges, and both compete with other disciplines for resources within their own institutions. As CM student numbers are not large in either Hong Kong or Australia, any fluctuations in enrolments caused by policy changes or increased competition would render these university programs vulnerable. In Australia, it is unlikely that new university programs will open in the near future, when universities teaching complementary medicine are under the scrutiny of sceptic groups that target universities' reputations [49]. Private college degree programs in Australia must go through a similar accreditation process as in universities, yet colleges are less likely to have access to the same research facilities and high-tech biomedical teaching equipment available in universities. A move towards greater private provision therefore may impact how CM is learned as well as the potential relationship between CM teaching and research.

Research provides another important source of income for universities, but, in both Hong Kong and Australia, research funding for CM is limited and not always funnelled into the same schools that actually teach CM. The research funding that is available for CM is typically directed mostly to basic science or clinical research involving standardised protocols. In general, research fitting a biomedical model attracts the largest funds and produces the most outputs for universities [28]. This factor, coupled with ongoing pressure for CM to “prove” its legitimacy within a context in which biomedicine dominates, means that within universities CM is likely to continue to become biomedicalised, at least when it comes to research, that is, tested through methods that do not necessarily allow for traditional knowledge or individualised approaches to be incorporated. Whether this biomedicalisation extends to how CM is taught within degree programs depends in part on how the relationship between teaching and research evolves, but a lack of alignment between the two domains is unlikely to be tenable in an environment where the scientific basis of CM degrees is under scrutiny. This points to an urgent need to evaluate the balance between research and education in the tertiary CM education sector.

A third important observation is that the relationship with mainland China exerts a significant influence on CM and CM education in both Australia and Hong Kong. This is interesting given that CM has been observed to have changed and adapted to the various transnational settings in which it is found [3, 55] and, in the case of Australia, the extensive period in which it has been established in local universities. Still, for both Australian and Hong Kong students, mainland China remains a common clinical training destination. This is despite the more restricted scope of practice and position in the health system for CM in these regions compared to the mainland. However, in Hong Kong, the recent policy developments and establishment of a CM hospital signify increased alignment with the status of CM in mainland China. For Australia, China already provides a useful source of CM research funding, and the new formal agreement between the two countries around CM means such investments are likely to continue. This may help to consolidate CM's position in Australian universities, although, as discussed, strengthened research programs will not necessarily impact the position of taught courses.

## 5. Conclusion

This cross-regional comparison has proved fruitful for identifying factors currently influencing the status of CM education, those that lie within and those that transcend national boundaries. The research has highlighted similarities and differences in CM education in Australia and Hong Kong in terms of history, current context and position, regulatory context and place in healthcare systems, and public and professional legitimacy. Further, the paper identifies issues of significance which have the potential to influence CM education in other regions, such as the impact of continued biomedical dominance within healthcare systems, and the increasing level of global competition between universities. Additionally, the relationship* between* nations has been identified as an important factor. While this currently revolves around links between mainland China and other regions, the global movement of the CM workforce may see connections developing between other regions in relation to CM and CM education. The growing worldwide popularity of CM and the associated demand for quality education programs underscores the relevance of this paper and highlights the necessity for future research into how the developments identified here might further impact the evolution of CM education.
